# Tissue versus mechanical mitral valve replacement in patients aged 50–70: a propensity-matched analysis

**DOI:** 10.1093/ejcts/ezae283

**Published:** 2024-08-21

**Authors:** Nicholas M Fialka, Abeline R Watkins, Abrar Alam, Ryaan EL-Andari, Jimmy J H Kang, Yongzhe Hong, Sabin J Bozso, Michael C Moon, Jeevan Nagendran

**Affiliations:** Division of Cardiac Surgery, Department of Surgery, University of Alberta, Edmonton, AB, Canada; Faculty of Medicine and Dentistry, University of Alberta, Edmonton, AB, Canada; Faculty of Medicine and Dentistry, University of Alberta, Edmonton, AB, Canada; Division of Cardiac Surgery, Department of Surgery, University of Alberta, Edmonton, AB, Canada; Division of Cardiac Surgery, Department of Surgery, University of Alberta, Edmonton, AB, Canada; Division of Cardiac Surgery, Department of Surgery, University of Alberta, Edmonton, AB, Canada; Division of Cardiac Surgery, Department of Surgery, University of Alberta, Edmonton, AB, Canada; Division of Cardiac Surgery, Department of Surgery, University of Alberta, Edmonton, AB, Canada; Division of Cardiac Surgery, Department of Surgery, University of Alberta, Edmonton, AB, Canada

**Keywords:** Mitral valve replacement, Bioprosthetic, Mechanical

## Abstract

**OBJECTIVES:**

There remains debate over the optimal mitral valve replacement (MVR) option for patients aged 50–70 years. The objective of this study was to retrospectively compare the long-term outcomes of mechanical and bioprosthetic MVR in this patient population.

**METHODS:**

Data from patients undergoing MVR between 2004 and 2018 were retrospectively reviewed. The primary outcome was all-cause mortality. Secondary outcomes included perioperative and late morbidity.

**RESULTS:**

Two hundred and eight-six propensity-matched patients (*n* = 143 mechanical; *n* = 143 bioprosthetic) aged 50–70 years were included in the final analysis. Maximum follow-up was 15.8 years. There was no significant difference in all-cause mortality between the groups at 30 days, 1 year, 5 years, 10 years, and at the longest follow-up. Patients who underwent mechanical MVR experienced significantly lower rates of postoperative atrial fibrillation (*P* = 0.001). There were no significant differences in rates of sepsis, acute kidney injury, superficial and deep sternal wound infection, mediastinal bleeding, and permanent pacemaker implantation. At the longest follow-up, there were no differences in myocardial infarction, stroke, heart failure or overall rehospitalization. At the same time point, there was an increased rate of MVR in patients receiving a bioprosthetic valve (*P* = 0.015).

**CONCLUSIONS:**

Survival following mechanical and bioprosthetic MVR in patients 50–70 years of age is similar to up to 15 years of follow-up. Bioprosthetic MVR is associated with an increased risk of repeat MVR. Mechanical MVR is not associated with an increased risk of stroke. Valve selection in this patient population requires diligent consideration of structural valve deterioration and subsequent reoperation risk as well as bleeding and thromboembolic risk.

## Graphical abstract

**Figure:**
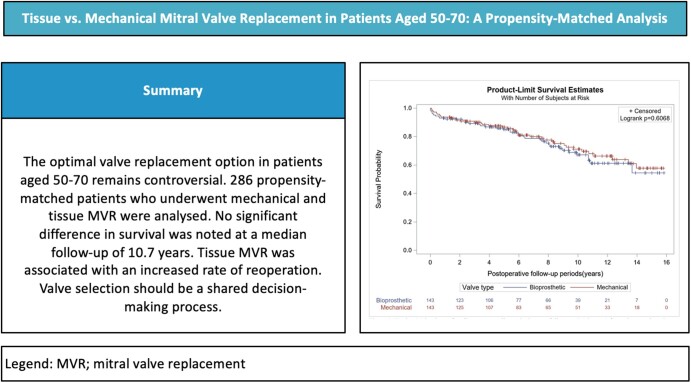

## INTRODUCTION

With a global prevalence of 2.5%, mitral valve (MV) disease remains the most common form of valvular heart disease and has a positive age-dependent association [1, 2]. The aetiology and presentation of MV disease vary considerably. Rheumatic fever, myxomatous degeneration, annular dilation and infective endocarditis all afflict the MV. The role of medical therapy is limited in progressive MV disease. Surgical intervention remains the gold standard of treatment for patients with severe, symptomatic mitral stenosis (MS) not amenable to percutaneous mitral balloon commissurotomy; as well as severe, symptomative mitral regurgitation. MV replacement (MVR) in particular is indicated when MV repair is not feasible [3].

Currently, 2 major forms of prosthetic heart valves are available—mechanical and bioprosthetic. Despite its long-term durability, the thrombogenicity of mechanical valves necessitates lifelong anticoagulation, subsequently introducing the risk of life-threatening haemorrhage. Conversely, bioprosthetic valves, commonly of bovine or porcine origin, offer excellent haemodynamic function without the need for permanent anticoagulation. However, spearheaded by a chronic xenogenic immune response, bioprostheses are prone to structural valve deterioration (SVD) often prompting reintervention [4–6]. Deterioration in younger patients translates into multiple repeat valve interventions throughout their lives. Therefore, contemporary American guidelines recommend the use of a mechanical prosthesis <65 years of age and a bioprosthesis in patients ≥65 years of age (Class 2a) [3]. Similarly, the 2021 ESC/EACTS guidelines on valvular heart disease recommend implantation of a mechanical valve and bioprosthetic valve in the mitral position in patients <65 years of age and >70 years of age, respectively (Class IIa; level of evidence B) [7].

The above recommendations have been formed largely on the basis of valve durability and subsequent risk of MV reintervention as well as long-term survival. The recommendations for mechanical MVR in younger patients (<50 years) and bioprosthetic MVR in older patients (>65–70 years) are clear and well-supported in the literature; however, there remains a grey, intermediary age group (50–70 years) in which an equivalent consensus on the superior prosthesis is lacking. The outcomes of mechanical vs bioprosthetic MVR in this patient population are highly pertinent and intriguing. Although the majority of the literature suggests improved long-term survival with mechanical MVR [8–12], there have been conflicting results [4, 13, 14], and MV aetiology appears to be a major factor at play [15, 16]. In an attempt to provide clarity to this discussion, we sought to retrospectively compare the outcomes of mechanical and bioprosthetic MVR in patients 50–70 years of age at our institution over a 15-year period.

## PATIENTS AND METHODS

### Study design

All Alberta residents undergoing surgical MVR at the Mazankowski Alberta Heart Institute between 1 January 2004 and 31 December 2018 were considered for inclusion in this retrospective cohort study. Preoperative, intraoperative and postoperative data was extracted from the APPROACH (Alberta Provincial Project for Outcome Assessment in Coronary Heart Disease) database, electronic health records, Provincial Death Registry and discharge abstract databases. Follow-up data were collected from postoperative assessments performed at the University of Alberta Hospital. Previous descriptions of the APPROACH database have been published elsewhere.

### Ethical statement

This study was approved by the local research ethics board on 26 November 2020—ethics protocol ID Pro00106075.

### Outcomes and definitions

The primary outcome was all-cause mortality at 30 days, 1 year, 5 years, 10 years and longest follow-up (15.8 years; median 10.7 years). Secondary outcomes consisted of various measures of perioperative and late morbidity. For the former, rates of new-onset atrial fibrillation (AF), sepsis, acute kidney injury (AKI), superficial sternal infection (SSWI), deep sternal infection (DSWI), clinically significant mediastinal bleeding and permanent pacemaker insertion (PPI) were evaluated. Measures of late morbidity included rates of hospitalization for myocardial infarction (MI), stroke, and heart failure (HF), as well as rates of overall rehospitalization, and MVR reoperation.

New-onset AF was defined as the ECG-documented occurrence of AF during the perioperative period in the absence of pre-existing AF. AKI was defined according to the Kidney Disease Improving Global Outcomes criteria of serum creatinine >1.5–1.9 baseline or ≥26 μmol increase. The diagnosis of superficial and deep sternal wound infections was clinical. Clinically significant mediastinal bleeding was defined as the return to the operating room for bleeding or need for blood transfusion. MI was defined as ST-segment elevation MI or non-ST-segment elevation MI. HF was defined as HF with reduced ejection fraction or HF with preserved ejection fraction. Both MI and HF were defined as a principal diagnosis upon hospital readmission. Reoperation was defined as repeat MVR or repair of complications originating from the original procedure.

### Statistical analysis

Continuous variables were reported as mean ± standard deviation or median (interquartile range) if not normally distributed. Categorical variables were summarized as count (%). Propensity score matching methods were applied to control for the difference in the baseline covariates. The propensity score was estimated using a multivariable logistic regression model with implanted MV type as the dependent variable and the baseline characteristics as covariates including age, body mass index, Society of Thoracic Surgeons score, sex, pulmonary disease, cerebrovascular disease, renal insufficiency, current smoker, former smoker, hypertension, dyslipidaemia, malignancy in 5 years, peripheral vascular disease, diabetes mellitus, congestive HF, AF, prior MI, prior percutaneous coronary intervention, prior coronary artery bypass grafting, and Concomitant procedures. Greedy matching techniques without replacement and a calliper width equal to 0.1 of the standard deviation of the logit of the propensity score were applied to match patients with bioprosthetic valves to patients with mechanical valves. Standardized mean difference was used to evaluate the balance before and after matching. A standardized difference of 0.1 or less was deemed to be the ideal balance and 0.1–0.2 was considered acceptable balance. Completeness of follow-up was determined using a completeness index using the ratio of the total observed follow-up as a percentage of the potential time of follow-up [17].

For in-hospital outcomes, the Wilcoxon signed-rank test was used to compare continuous variables while categorical variables were compared with McNemar’s test. Cox proportional hazards regression models and Fine & Gray model were adopted to estimate the hazard ratios of the difference between the primary and secondary outcomes. The survival curve was plotted for all-cause mortality at the longest follow-up using Kaplan–Meier methods. Statistical analyses were executed using the SAS 9.4 (SAS Institute, Cary, NC). A *P*-value <0.05 was deemed statistically significant. All statistical tests were 2-sided.

## RESULTS

### Study cohort

**Figure 1:**
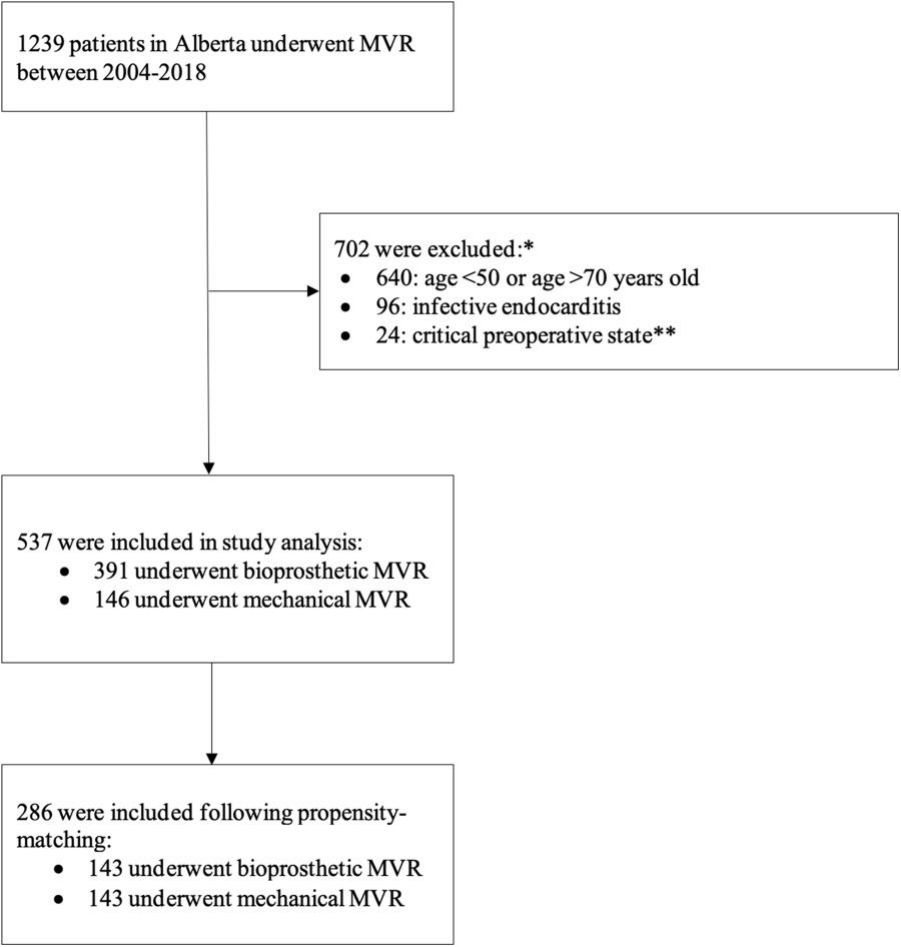
Study population flowchart. *Some overlap existed; therefore, the numbers breakdown did not add up to the total. **Need for intra-aortic balloon pump, respiratory failure requiring vent, cardiogenic shock. MVR: mitral valve replacement.

Initially 1239 patients were identified. Patients were excluded if their age was < 50 or > 70 (*n* = 702), they were diagnosed with infective endocarditis (*n* = 96), or they were in a critical preoperative state (*n* = 24; Fig. 1), resulting in 537 patients meeting the inclusion criteria. Following propensity-matching, 286 patients were included in the final analysis (*n* = 143 mechanical; *n* = 143 bioprosthetic).

### Baseline demographics

Baseline characteristics of the overall and propensity-matched patient populations are included in Table 1. Prior to propensity-matching, patients who received bioprosthetic valves were older (62.1 ± 5.3 vs 58.2 ± 5.3; standardized mean difference 0.73), and more commonly underwent concomitant coronary artery bypass grafting (33.8% vs 17.1%; standardized mean difference 0.39) or ascending aortic replacement (4.1% vs 0.7%; standardized mean difference 0.22). No significant differences were noted between the matched groups. Regarding MV pathology, there was a similar distribution in disease aetiology in patients undergoing mechanical and bioprosthetic MVR. Of note, approximately half of all patients suffered from rheumatic heart disease (Supplementary Material, Table S1).

**Table 1:** Baseline characteristics of patients undergoing mitral valve replacement

	Before matching			After matching		
Characteristics	Mechanical (*n* = 146)	Bioprosthetic (*n* = 391)	Standardized difference	Mechanical (*n* = 135)	Bioprosthetic (*n* = 135)	Standardized difference
Age (years)	58.2 ± 5.3	62.1 ± 5.3	0.73	58.4 ± 5.3	58.8 ± 5.2	0.08
BMI (kg/m^2^)	29.2 ± 6.1	29 ± 6.6	0.02	29.3 ± 6.1	29.4 ± 6.8	0.01
STS score	1.6 ± 0.7	2.1 ± 1.2	0.53	1.6 ± 0.7	1.6 ± 0.6	0.08
Male	64 (43.8)	186 (47.6)	0.08	62 (43.4)	64 (44.8)	0.03
COPD	73 (50)	208 (53.2)	0.06	73 (51)	75 (52.4)	0.03
CEVD	13 (8.9)	48 (12.3)	0.11	13 (9.1)	14 (9.8)	0.02
Renal insufficiency	13 (8.9)	55 (14.1)	0.16	13 (9.1)	14 (9.8)	0.02
Current smoker	44 (30.1)	136 (34.8)	0.10	44 (30.8)	45 (31.5)	0.02
Former smoker	53 (36.3)	152 (38.9)	0.05	51 (35.7)	55 (38.5)	0.06
Hypertension	90 (61.6)	269 (68.8)	0.15	89 (62.2)	96 (67.1)	0.1
Dyslipidemia	101 (69.2)	298 (76.2)	0.16	98 (68.5)	98 (68.5)	<0.01
Malignancy in previous 5 years	2 (1.4)	12 (3.1)	0.12	2 (1.4)	2 (1.4)	<0.01
PAD	4 (2.7)	14 (3.6)	0.05	4 (2.8)	6 (4.2)	0.08
Diabetes	31 (21.2)	102 (26.1)	0.11	31 (21.7)	29 (20.3)	0.03
Heart failure	62 (42.5)	201 (51.4)	0.18	61 (42.7)	64 (44.8)	0.04
Atrial fibrillation	14 (9.6)	58 (14.8)	0.16	14 (9.8)	15 (10.5)	0.02
Prior history of MI	12 (8.2)	42 (10.7)	0.09	12 (8.4)	11 (7.7)	0.03
Prior history of PCI	4 (2.7)	8 (2)	0.05	4 (2.8)	5 (3.5)	0.04
Prior history of CABG	9 (6.2)	22 (5.6)	0.02	8 (5.6)	7 (4.9)	0.03
Concomitant procedures						
CABG	25 (17.1)	132 (33.8)	0.39	24 (16.8)	18 (12.6)	0.12
AVR or AVr	25 (17.1)	92 (23.5)	0.16	24 (16.8)	24 (16.8)	<0.01
TVR or TVr	54 (37)	137 (35)	0.04	51 (35.7)	55 (38.5)	0.06
Aortic root replacement	2 (1.4)	12 (3.1)	0.12	2 (1.4)	2 (1.4)	<0.01
Ascending aortic replacement	1 (0.7)	16 (4.1)	0.22	1 (0.7)	1 (0.7)	<0.01

AVr: aortic valve repair; AVR: aortic valve replacement; BMI: body mass index; CABG: coronary artery bypass grafting; CEVD: cerebrovascular disease; COPD: chronic obstructive pulmonary disease; MI: myocardial infarction; PAD: peripheral artery disease; PCI: percutaneous coronary intervention; STS: Society of Thoracic Surgery; TVr: tricuspid valve repair; TVR: tricuspid valve replacement.

### Intraoperative characteristics

There was no significant difference in cardiopulmonary bypass (CPB) time (median 145 vs 155 min; *P* = 0.185), aortic cross-clamp time (median 115 vs 121 min; *P* = 0.144), or overall procedure time (median 4 vs 4 h; *P* = 0.375) between patients receiving mechanical and bioprosthetic valves, respectively (Table 3).

**Table 3:** Procedural and in-hospital outcomes of patients undergoing mitral valve replacement in the propensity-matched cohort

Outcome	Mechanical (*n* = 143)	Bioprosthetic (*n* = 143)	*P*-value
Operative parameters			
CPB time, min, median (q1–q3)	145 (120–192.5)	155 (127–212)	0.185
AoCX time, min, median (q1–q3)	115 (87–155)	121 (100–165)	0.144
Procedure time, h, median (q1–q3)	4 (3–6)	4 (3–6)	0.375
Perioperative morbidity			
New-onset atrial fibrillation	17 (11.9%)	39 (27.3%)	**0.001**
Sepsis	4 (2.9%)	7 (5.1%)	0.366
Acute kidney injury	6 (4.3%)	12 (8.7%)	0.157
Superficial sternal infection	8 (5.7%)	8 (5.7%)	>0.999
Deep sternal infection	2 (1.4%)	3 (2.2%)	0.655
Mediastinal bleeding	2 (1.4%)	1 (0.7%)	0.564
Permanent pacemaker insertion	6 (4.3%)	5 (3.6%)	0.738

AoCX: aortic cross-clamp; CPB: cardiopulmonary bypass. Bolded values indicate statistical significance.

### Primary outcome

**Figure 2:**
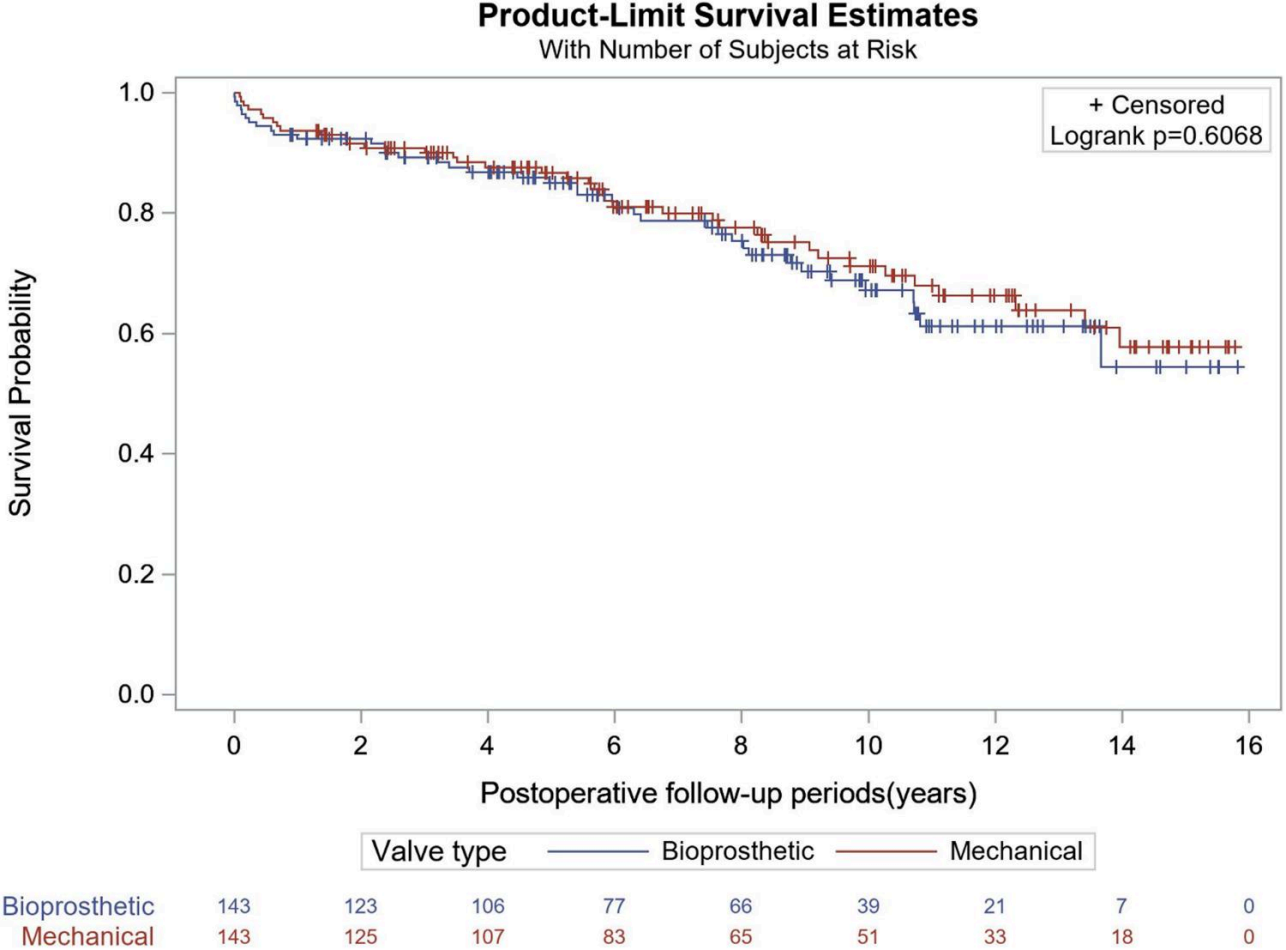
Kaplan–Meier curve for all-cause mortality at the longest follow-up (15.8 years) in patients undergoing mechanical versus bioprosthetic mitral valve replacement.

The primary outcome is summarized in Table 2. There was no significant difference in all-cause mortality at 30 days (0 vs 2.1%), 1 year (6.3 vs 7.7%; *P* = 0.628), 5 years (12.6 vs 14%; *P* = 0.695), 10 years (22.4 vs 14.5%; *P* = 0.593), and at the longest available follow-up (26.9 vs 27.3%; *P* = 0.607; median follow-up 10.7 years). Kaplan–Meier curve indicated equivalent all-cause mortality up to 15.8 years (*P* = 0.6068; Fig. 2). Follow-up was 99.2%, 95.3%, 82.3%, 65.3% and 46.6% at 30 days, 1 year, 5 years, 10 years, and the longest available follow-up, respectively.

**Table 2:** Primary outcomes of patients undergoing mitral valve replacement in the propensity-matched cohort

Mortality	Median follow-up time	Bioprosthetic (*n* = 143)	Mechanical (*n* = 143)	Hazard ratio^a^ (95% CI)	*P*-value
Death at 30 days (%)	30 days	3 (2.1)	0 (0)	NA	NA
Death at 1 year (%)	12 months	11 (7.7)	9 (6.3)	1.24 (0.52–3.00)	0.628
Death at 5 years (%)	5 years	20 (14)	18 (12.6)	1.14 (0.6–2.15)	0.695
Death at 10 years (%)	9.4 years	35 (14.5)	32 (22.4)	1.14 (0.71–1.84)	0.593
Death at longest follow-up (%)	10 years	39 (27.3)	38 (26.9)	1.12 (0.72–1.76)	0.607

a Reference group is the mechanical valve group. NA, not applicable due to quesi-seperation (zero in numerator or denominator). Longest follow-up was 15.8 years.

### Secondary outcomes

Patients who underwent mechanical MVR experienced significantly lower rates of postoperative AF (11.9 vs 27.3%; *P* = 0.001). No statistically significant differences in the rates of sepsis (2.9 vs 5.1%; *P* = 0.366), AKI (4.3 vs 8.7%; *P* = 0.157), SSWI (5.7 vs 5.7%; *P* > 0.999), DSWI (1.4 vs 2.2%; *P* = 0.655), mediastinal bleeding (1.4 vs 0.7%; *P* = 0.564) and PPI (4.3 vs 3.6%; *P* = 0.738) were present between the groups. There were no significant differences in rehospitalization for MI (2.8 vs 0.7%; *P* = 0.208), stroke (5.6 vs 7.7%; *P* = 0.582), HF (39.9 vs 29.4%; *P* = 0.100) or overall rehospitalization (63.6 vs 59.4%; *P* = 0.475) at a median follow-up of 10.7, 10.7, 9.7, and 9.4 years, respectively. At a median follow-up of 10.7 years, there was an increased rate of MVR in patients receiving a bioprosthetic valve (9.1 vs 2.0%; *P* = 0.015; Table 4). The cumulative incidence plots for the secondary outcomes have been included in Supplementary Material, Fig. S1. Follow-up was 52.9%, 51.9%, 37.6%, 21.3% and 44.7% for MI, stroke, HF, overall hospitalization, and redo-MVR, respectively.

**Table 4:** Long-term morbidity of patients undergoing mitral valve replacement in the propensity-matched cohort

Outcome	Median follow-up time (years)	Bioprosthetic (*n* = 143)	Mechanical (*n* = 143)	Hazard ratio^a^ (95% CI)	*P*-value
Hospitalization for MI at longest follow-up (%)	10.7	4 (2.8)	1 (0.7)	4.1 (0.45–37.4)	0.208
Hospitalization for Stroke at longest follow-up (%)	10.7	8 (5.6)	11 (7.7)	0.78 (0.32–1.89)	0.582
Hospitalization for HF at longest follow-up (%)	9.7	57 (39.9)	42 (29.4)	1.40 (0.94–2.09)	0.100
Re-hospitalization at longest follow-up (%)	9.4	91 (63.6)	85 (59.4)	1.11 (0.83–1.49)	0.475
Redo MVR at longest follow-up (%)	10.7	13 (9.1)	3 (2)	4.8 (1.36–17.2)	**0.015**

a Reference group is the mechanical valve group. NA, not applicable due to quesi-seperation (zero in numerator or denominator). MI: myocardial infarction; HF: heart failure; MVR: mitral valve replacement. Longest follow-up was 15.8 years. Bold values indicate statistical significance.

## DISCUSSION

There are several notable findings in this retrospective, single-centre analysis. First and foremost, survival following mechanical and bioprosthetic MVR in patients 50–70 years of age is similar not only in the perioperative period but also at a median follow-up of 10.7 years (Table 2; Fig. 2). Furthermore, perioperative morbidity was largely equivalent between the groups, with similar rates of sepsis, AKI, SSWI, DSWI, mediastinal bleeding and PPI; however, patients undergoing bioprosthetic MVR were more likely to experience postoperative AF (Table 3). At the longest follow-up, patients undergoing bioprosthetic MVR experienced increased rates of MV reoperation. Long-term rates of stroke, MI, and HF were similar between the groups.

The have been 2 primary composite analyses on the topic to date. In their meta-analysis of 14 studies and >20 000 patients, Yanagawa *et al.* reported significantly lower operative mortality, long-term mortality (median 8 years), and MV reoperation following mechanical MVR. Conversely, rates of bleeding were significantly lower with bioprosthetic MVR, along with a trend towards a reduced risk of stroke and embolism [11]. Similarly, Ahmed *et al.* indicated lower 30-day and long-term (mean 14.1 years) mortality with mechanical MVR. Rates of MV reoperation, stroke, and bleeding were equivalent [10]. Evidently, the equivalent long-term mortality observed in the present analysis contrasts with these previous results. It is important to note that both aggregate analyses included studies with patients <70 years of age, without a lower age limit. In the composite analysis by Ahmed *et al.* in particular the mean age ranged from 46.5 to 63.0 years old [10]. Consequently, the target population of 50–70-year-olds in which the optimal prosthesis choice is still controversial was not selectively analysed and younger patients (<50) in whom SVD is accelerated, potentially confounded these results [18].

It has been postulated that the improved survival following mechanical MVR as compared to bioprosthetic MVR is a product of the larger effective orifice area of mechanical valves, and subsequently reduced transvalvular pressure gradients promoting greater positive cardiac remodelling [11, 19]. There does remain a paucity of literature analysing haemodynamic/myocardial remodelling following mechanical and bioprosthetic MVR. This could help delineate the differences observed in previous analyses [12]. The considerable morbidity and mortality associated with MV reoperation may also contribute to inferior long-term mortality following bioprosthetic MVR [20]. Finally, patients undergoing bioprosthetic MVR may possess an increased perioperative risk profile (i.e. infective endocarditis) [21]. Subgroup analysis of patients with infective endocarditis in the aforementioned meta-analysis by Ahmed *et al.* indicated significantly superior late survival with mechanical MVR [10]. Herein may lie an additional factor underlying the equivalent long-term mortality in the present analysis as patients with infective endocarditis were excluded.

Increased rates of MV reoperation in patients receiving bioprosthetic valves are consistent with previous literature. SVD, multifactorial in aetiology with a chronic xenogenic immune response, dystrophic calcification, and mechanical stress all contributing, limits the durability of tissue valves [5, 6]. Moreover, differences observed in rates of new-onset AF may be due to a variety of factors. For one, modifications in institutional practice targeted at reduced postoperative AF including consistent prophylactic beta-blockade and strict electrolyte management, have occurred over the ∼2 decades of data collection [22]. Additionally, risk factors for postoperative AF, including left ventricular dysfunction, left atrial enlargement and β-blocker withdrawal are not all included in the baseline characteristics and controlled for [23, 24]. It should be noted that perioperative AF has been associated with an increased risk of stroke and mortality at mid-late follow-up. Underprescription of anticoagulation in favour of reducing perioperative and long-term bleeding risk likely contributes to these outcomes [25, 26]. Interestingly, this did not translate to inferior survival in patients undergoing bioprosthetic MVR in this study.

The treatment of valvular heart disease is a highly innovative and rapidly expanding field. Advancements in both mechanical and tissue valves over the last few decades have been instrumental in improving the outcomes of this patient population as a whole. Given the attractive advantages of a bioprosthesis, considerable research has been targeted at improving the durability of these valves. The advent of transcatheter MV therapies may additionally provide patients with a less invasive option in the redo-setting [27]. However, advancements in mechanical valve technology have also been made. Thromboembolism and/or bleeding remain the primary causes of morbidity with mechanical prostheses. Optimal anticoagulation and international normalized ratio (INR) targets for this patient population have recently been examined in the Prospective Randomized On-X Anticoagulation Trial (PROACT) trials. Although the results of the PROACT Mitral trial failed to demonstrate the noninferiority of the composite primary end-point of thromboembolism, valve thrombosis, or bleeding in patients receiving low-dose warfarin (INR 2.0–2.5) vs standard-dose warfarin (INR 2.5–3.5), this trial was limited by a considerable overlap in INR between the groups [28]. Finally, improvements in perioperative care and the feasibility of INR testing at home and/or via telemedicine reduce morbidity and have the potential to improve the quality of life in patients with mechanical valves [11, 29, 30].

Ultimately valve selection is multifactorial and must be patient-centred, with shared decision-making the hallmark of navigating these discussions (ACC/AHA Class I recommendation) [3]. Consideration of pre-existing indications for anticoagulation, SVD, projected life expectancy, bleeding/stroke risk, medication compliance, social situation, as well as patient preference is paramount. The present results contribute to the growing body of literature on prosthesis selection in MVR and should help guide patients and physicians in making individualized, informed decisions.

### Limitations

This study does come with limitations. For one, despite our attempts to mitigate selection bias with propensity-matching patients based on baseline and operative characteristics, the retrospective nature of the analysis has inherent drawbacks. Additionally, the sample size of the final analysis limits statistical power. Consequently, we have not conducted a subanalysis based on decade (patients 50–60 years of age vs patients 60–70 years of age). The 50–70 age range was chosen to encompass the contemporary American and European guidelines on valve selection. Similarly, we were unable to identify risk factors/factors associated with early SVD that are driving the increased reintervention rates in the bioprosthetic group. Long-term follow-up varied according to the outcome analysed, ranging from 21.3% to 52.9%. However, the completeness index utilized to demonstrate the completeness of follow-up is greatly influenced by the event rate. This also further highlights a limitation of the retrospective study design. Additionally, the limits of our database prohibited analysis of major bleeding events and infective endocarditis, pertinent outcomes to the comparison of bioprosthetic and mechanical valve replacement. Data regarding the cause of death were similarly unavailable.

## CONCLUSION

We provide a retrospective, single-centre analysis of bioprosthetic versus mechanical MVR in patients 50–70 years of age. Survival following mechanical and bioprosthetic MVR in patients 50–70 years of age is similar at a median follow-up of 10.7 years. Perioperative morbidity was comparable between the groups, with similar rates of sepsis, AKI, SSWI, DSWI, mediastinal bleeding, and PPI; however, patients undergoing bioprosthetic MVR experienced increased rates of postoperative AF as well as MV reoperation in the long-term. Mechanical MVR was not associated with an increased risk of late stroke. Based on these results, valve selection in this patient population should not be based on mortality alone; but rather requires diligent consideration of SVD and subsequent reoperation risk as well as bleeding and thromboembolic risk.

## Supplementary Material

ezae283_Supplementary_Data

## Data Availability

The data underlying this article are available in the article and in its online supplementary material.

## ABBREVIATIONS

AF: Atrial fibrillation
AKI: Acute kidney injury
APPROACH: Alberta Provincial Project for Outcome Assessment in Coronary Heart Disease
DSWI: Deep sternal infection
HF: Heart failure
MI: Myocardial infarction
MS: Mitral stenosis
MV: Mitral valve
MVR: Mitral valve replacement
PPI: Permanent pacemaker insertion
PROACT: Prospective Randomized On-X Anticoagulation Trial
SSWI: Superficial sternal infection
SVD: Structural valve deterioration
