# Supplementary material for: Tissue versus mechanical mitral valve replacement in patients aged 50–70: a propensity-matched analysis
Source: Eur J Cardiothorac Surg. 2024 Aug 21;66(2):ezae283. doi: 10.1093/ejcts/ezae283 (PMC11344592; doi:10.1093/ejcts/ezae283)
Supplement: ezae283_Supplementary_Data [file ezae283_supplementary_data.docx]

Supplementary Table S1. Etiology of mitral valve disease in patients undergoing mitral valve replacement.

| **Etiology** | **Bioprosthetic (n=134)** | **Mechanical (n=133)** |
| --- | --- | --- |
| Annular calcification | 11 (8.2%) | 20 (15%) |
| Prosthetic valve dysfunction | 32 (23.9%) | 22 (16.5%) |
| Rheumatic | 63 (47%) | 63 (47.4%) |
| Annular dilation | 3 (2.2%) | 7 (5.3%) |
| Ischemic | 1 (0.7%) | 4 (3%) |
| Congenital | 1 (0.7% | 0 (0%) |
| Degenerative | 25 (18.7%) | 22 (16.5%) |
| Other | 16 (11.9%) | 10 (7.5%) |


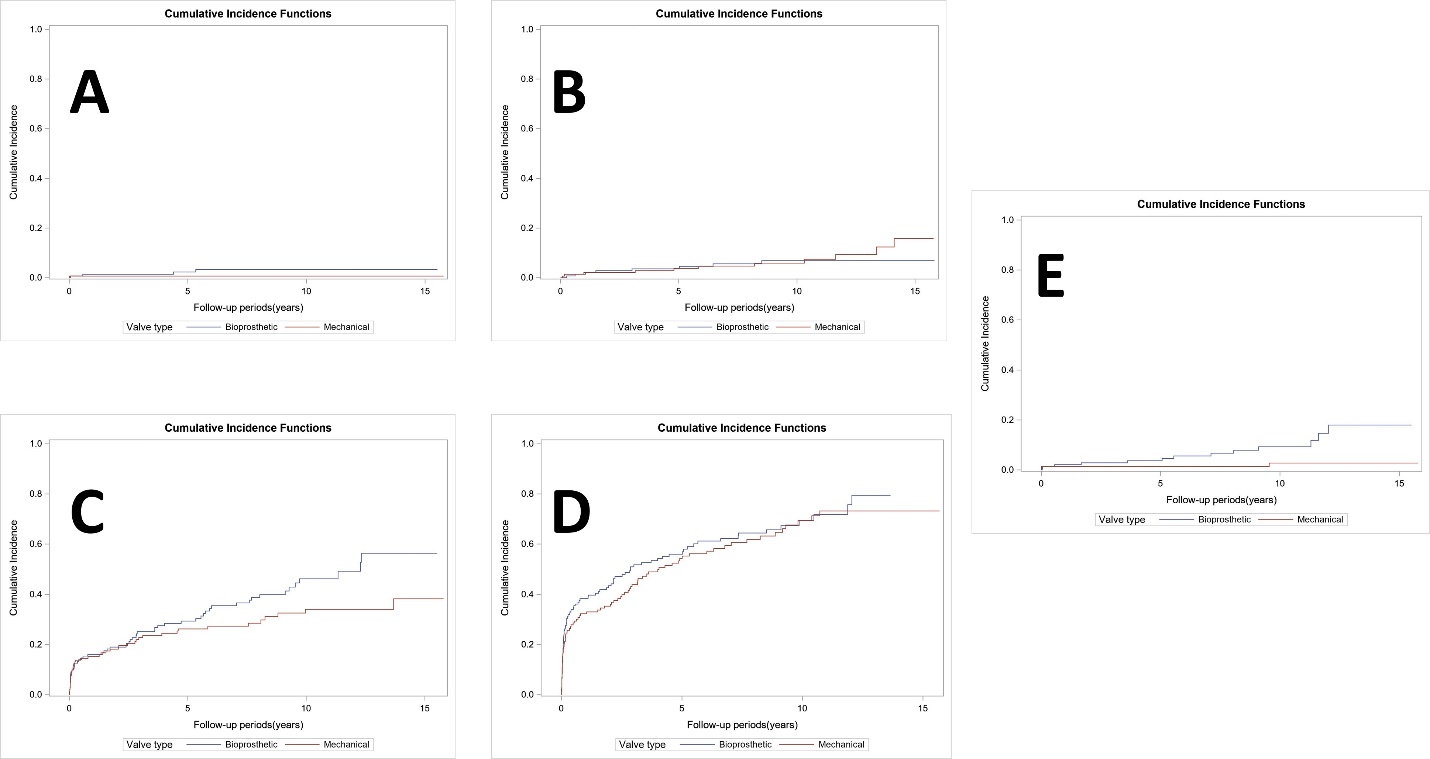


Supplementary Figure S1. Cumulative incidence curve for (A) myocardial infarction, (B) stroke, (C) readmission for heart failure, (D) overall readmission, (E) redo mitral valve replacement.
